# Distribution and quantitative changes in amounts of aquaporin 1, 5 and 9 in the pig uterus during the estrous cycle and early pregnancy

**DOI:** 10.1186/1477-7827-8-109

**Published:** 2010-09-09

**Authors:** Mariusz T Skowronski

**Affiliations:** 1 Department of Animal Physiology, University of Warmia and Mazury, Olsztyn, Poland

## Abstract

**Background:**

Aquaporins (AQPs) are a family of membrane channel proteins that facilitate bulk water transport. To date, 11 isoforms of AQPs have been reported to be expressed in the female and male reproductive systems. The purpose of our study was to determine the localization and quantitative changes in the expression of AQP1, 5 and 9 within the pig uterus during different stages of the estrous cycle and early pregnancy.

**Methods:**

Immunoperoxidase and semi-quantitative immunoblotting techniques were used to examine the distribution and changes in amounts of AQP1, AQP5 and AQP9 in uteral cells of pigs at the early (Days 2-4), middle (10-12), late (14-16) stage of the luteal phase and late (18-20) stage of the follicular phase of the estrous cycle as well as on Days 14-16 and 30-32 of gestation (the onset and the end of implantation process).

**Results:**

The results demonstrated that AQP1, 5, and 9 were clearly detected in all studied stages of the estrous cycle and pregnancy. AQP1 was localized within uterine blood vessels. In cyclic gilts, endometrial and myometrial expression of AQP1 protein did not change significantly but increased during gestation. AQP5 was localized in smooth muscle cells and uterine epithelial cells. Endometrial expression of AQP5 protein did not change significantly between Days 2-4 and 10-12 of the estrous cycle but increased on Days 14-16 and 18-20 as well as during early pregnancy. Myometrial expression of AQP5 did not differ significantly during the estrous cycle but increased in the pregnancy. The anti-AQP9 antibody labeled uterine epithelial cells of uterus. Endometrial expression of AQP9 did not change significantly between Days 2-4 and 10-12 of the estrous cycle but increased on Days 14-16 and 18-20 as well as during early pregnancy.

**Conclusions:**

The results suggest that a functional and distinctive collaboration exists among diverse AQPs in water handling during the different uterine phases in the estrous cycle and early pregnancy.

## Background

Aquaporins (AQPs) are integral plasma membrane proteins that primarily transport water across the plasma membrane. These proteins were identified more than a decade ago [1]. There are 13 members (AQP0-12) in humans and many other AQPs have also been found in plants, yeast, bacteria, amphibians, and various lower organisms [2]. Aquaporins, based on their structural and functional properties, are divided into three subgroups: classical aquaporins (AQP0, 1, 2, 4, 5, 6, 8), aquaglyceroporins (AQP3, 7, 9, 10), and recently identified, so called superaquaporins (AQP11, 12) (for review see [3]). Since specific inhibitors were not previously available, physiological roles of AQPs are suggested on the basis of experiments with AQP knock-out (KO) mice and humans. For example, abnormal water metabolism was shown with AQP1, 2, 3, 4, 5 KO mice, and abnormal glycerol transport was shown with AQP3, 7, 9 KO mice. AQP0, 1, 2, 3, 7 null humans have also been reported (for review see [4]). Based on the protein expression, so far at least nine AQP isoforms have been confirmed to be present in the female reproductive system of humans, rats and mice (for review see [5]).

The first confirmation of AQP in the female reproductive system was obtained by isolating and sequencing the complementary DNA (cDNA) encoding water channels from the human uterus [6]. Afterwards, Li et al. [7] found AQP1 mRNA in the rat uterus. Edashige et al. [8] showed the expression of AQP3 and AQP7 mRNA in mouse oocytes. The presence of AQP3 mRNA in mouse oocytes was recently confirmed by Meng et al. [9]. AQP7, 8 and/or 9 have been shown to participate in water influx across the ovarian follicle wall primarily through transcellular transport mechanisms in the rat [10]. Recently, Skowronski et al. [11] have detected AQP1 expression in the capillary endothelium, AQP5 in the flattened follicle cells of primordial follicles and in the granulosa cells of developing follicles as well as AQP9 in the granulosa cells. In literature, there have been three reports pertaining to AQP localization in oviductal tissues. Branes et al. [12] showed by immunohistochemistry the expression of AQP5, AQP8 and AQP9 in the epithelial cells of the rat oviduct. In turn, Gannon et al. [13] found AQP1 labeling in the inner cells of the circular muscular layer of the rat oviduct. Our resent work demonstrated the localization of AQP1 in the pig oviductal vessels, AQP5 in muscle cells as well as AQP5 and AQP9 in oviductal luminal epithelium [11]. In 2003, two independent groups found AQP1 expression in the mouse myometrium [14,15]. Lindsay & Murphy [16] reported AQP1 expression in endothelial cells of the endometrium and in the inner circular layer of the rat myometrium. The presence of AQP5 in the uterine epithelia has been demonstrated in ovarectomized [16] and pregnant rats [17] as well as in mice during implantation [15]. Lindsay & Murphy [17] showed AQP9 expression in the apical plasma membrane of the glandular epithelium of the rat uterus. Recently, Skowronski et al. [11] showed the expression of AQP1 in the endothelial cells of the uterine blood vessels, AQP5 in the myometrium, as well as AQP5 and AQP9 in luminal and glandular epithelium. Moreover, AQP1, AQP2, and AQP5 have been identified in the bitch uterus [18]. AQP2 expression was also found in human endometrial cells of the uterus [19,20]. There is also evidence indicating that ovarian steroids can affect the expression of several AQPs [12,14,16]. It suggests that marked fluctuations in AQP expression in target tissues for steroids, including the uterus, may exist. In our previous study, immunohistochemistry and Western blot analyses using antibodies against nine AQPs (AQP1, 2, 3, 4, 5, 7, 8, 9, and 11) were performed to examine whether these water channel proteins are expressed in the pig uterus on days 17-19 of the estrous cycle (follicular phase). The analysis confirmed the expression of AQP1, AQP5, and AQP9 [11]. Cellular localization of aquaporin isoforms in the mammalian uterus are presented in Table 1.

**Table 1:** Cellular localization of aquaporin isoforms in the mammalian uterus

Aquaporin	Permeable to water/glycerol	Expression pattern
AQP0	Low/-	NA
AQP1	High/-	Vs, MC [11,14-16,18,42,43,47,48]
AQP2	High/-	EP, MC [14,18-20]
AQP3	High/Yes	EP [14]
AQP4	High/-	EP [15]
AQP5	High/-	EP, MC [11,15-18,48]
AQP6	Low/-	NA
AQP7	High/Yes	NA
AQP8	High/-	ST, MC [14]
AQP9	Low/Yes	EP [11,17]
AQP10	Low/Yes	NA
AQP11	NA/NA	NA
AQP12	NA/NA	NA

EP - epithelial cells, MC - muscle cells, ST - stroma, Vs - vessels, NA - data are not available.

Nevertheless, data concerning the quantitative expression of AQPs in the uterus are still very limited and not available in relation to the pig. Therefore, the aim of this study was to examine the tissue and cellular localization as well as changes in protein expression of AQP1, 5 and 9 in the uterus of gilts during the estrous cycle and early pregnancy. Present results clearly demonstrated that AQP1, -5, and -9 protein expression in the porcine uterus is influenced by estrus cycle and pregnancy.

## Methods

### Reagents

In the present study, affinity-purified polyclonal antibodies to AQPs were used (SulfoLink Kit, Pierce). The antibodies to AQP1, AQP5 and AQP9 were previously characterized, respectively, by [21-23]. Moreover, the anti-β-actin antibody was used (cat. no. A2066, Sigma-Aldrich). In addition, immunoglobulins from non-immunized rabbit were used as a negative control.

### Animals

All experiments were performed in accordance with the principles and procedures of Animal Ethics Committee of the University of Warmia and Mazury in Olsztyn. Tissue samples were recovered from mature cross-bred gilts (Large White × Polish Landrace) at the early Days 2-4 (n = 5), middle 10-12 (n = 5), late 14-16 (n = 5) stage of the luteal phase and late 18-20 (n = 5) stage of the follicular phase of the estrous cycle as well as on Days 14-16 (n = 5) and 30-32 (n = 5) of gestation (the onset and the end of implantation process). Gilts were observed daily for estrus behavior in the estrous cycle, and they were used during their third consecutive normal estrous cycle. Additionally, stage of the cycle was confirmed according to [24]. Gilts assigned to the pregnant group were naturally bred on the second day of estrus. Endometrium was separated from myometrium in each gilts, and 10-g weight fragments of both tissue types were frozen in liquid nitrogen immediately after dissection and then stored at -70°C until Western blot analysis. For immunohistochemistry, tissues were fixed by immersion in 4% paraformaldehyde for 24 h [11].

### SDS-PAGE and Western blot

Following isolation, the tissues were immediately placed in ice-cold dissection buffer (0.3 M sucrose, 25 mM imidazol, 1 mM EDTA in ddH_2_O, pH 7.2) containing 8.4 μM leupeptin and 0.4 mM pefabloc [11]. The tissue samples were homogenized using an ultra Turrax T8 homogeniser (IKA Labortechnik, Staufen, Germany) and centrifuged at 4,000 × g for 15 min at 4°C. The supernatant diluted in SDS buffer contained a final concentration of 62 mM Tris (hydroxymethyl)-aminomethane, 0.1 M sodium dodecyl sulphate (SDS), 8.7% glycerol, 0.09 mM bromophenol blue and 0.04 M dithiothreitol (DTT), pH 6.8. The protein samples were heated for 5 min at 90°C and stored in refrigerator for further analysis.

The samples warmed up to 37°C were loaded into 12.5% polyacrylamide gels and proteins were separated by electrophoresis. The total protein amount in each sample was adjusted by staining with Gelcode Coomassie Blue Stain Reagent (Bie and Berntsen A/S, Åbyhøj, Denmark) in order to provide equal loading. The proteins of studied gels were then electro-transferred onto nitrocellulose membranes (Hybond ECL RPN3032 D, Amersham Pharmacia Biotech, Little Chalfont, UK) for 1 h at 100 V. The membranes were blocked with 5% milk in PBS-T (80 mM Na_2_HPO_4_, 20 mM NaH_2_PO_4_, 100 mM NaCl, pH 7.5 and 0.1% vol/vol Tween 20) for 1 h. After washing, the membranes were incubated overnight at 5°C with anti-AQPs, anti-AQPs preadsorbed with the excess synthetic peptide or β-actin antibodies.

The membranes were washed and incubated with horseradish peroxidase-conjugated goat anti-rabbit IgG secondary antibody (P448, diluted 1:3,000, Dako A/S, Glostrup, Denmark) in PBS-T for 1 h. After washing with PBS-T, the sites of antibody-antigen reaction were visualized with an enhanced chemiluminescence (ECL) system (Amersham Pharmacia Biotech, Little Chalfont, UK) and exposure to photographic film (Hyperfilm ECL, RPN3103K, Amersham Pharmacia Biotech, Little Chalfont, UK). The results of Western blotting were quantified by densitometric scanning of immunoblots with GelScan for Windows ver. 1.45 software (Kucharczyk, Poland). Data were expressed as a ratio of AQP proteins relative to actin protein in OD.

### Immunohistochemistry

For preparation of paraffin-embedded tissue sections (4-μm thickness), the tissues were dehydrated in ethanol followed by xylene and finally embedded in paraffin [25]. The staining was carried out using indirect immunoperoxidase. The sections were dewaxed and rehydrated. For immunoperoxidase labeling, endogenous peroxidase was blocked by 0.5% H_2_O_2 _in absolute methanol for 10 min at room temperature. To reveal antigens, the sections were submerged in 1 mM Tris solution (pH 9.0) supplemented with 0.5 mM EGTA and heated in a microwave oven. After the treatment, the sections were left for 30 min in the buffer for cooling. Nonspecific binding of IgG was eliminated by incubating the sections in 50 mM NH_4_Cl for 30 min, followed by blocking in PBS supplemented with 1% BSA, 0.05% saponin and 0.2% gelatin. The sections were incubated overnight at 4°C with primary antibodies, or primary antibodies preadsorbed with the excess synthetic peptides (described above), diluted in PBS supplemented with 0.1% BSA and 0.3% Triton X-100. The sections were rinsed with PBS supplemented with 0.1% BSA, 0.05% saponin and 0.2% gelatin, and then incubated with horseradish peroxidase-conjugated secondary antibody (Dako A/S, Glostrup, Denmark). Labeling was visualized by 0.05% 3,3 diaminobenzidine tetrahydrochloride (DAB). The microscopy was carried out using a Leica DMRE light microscope (Heidelberg, Germany).

### Reverse transcription - polymerase chain reaction (RT-PCR)

Total RNA from fresh tissues was extracted using RNeasy Mini Kits (Qiagen, Germantown, MD) [25]. After DNase-treatment (RQ1 RNase-Free DNase, Promega, Madison, WI), the RNA was reverse transcribed using 2 U/μl Reverse Transcriptase (Superscript II, Invitrogen, Taastrup, Denmark) in the presence of poly-T primers. RT products were stored at -20°C until required. Primers were designed to amplify specifically *AQPs *transcript: The following primers were used: *AQP1 *(221 bp product): Sense: 5-TTGGGCTGAGCATTGCCACGC-3; Antisense: 5-CAGCGAGTTCAGGCCAAGGGAGTT-3; *AQP5 *(466 bp product): Sense: 5-CCATCCTGCAGATCGCGCTAGC-3; Antisense: 5- ATGACGACTGCGGGGCCGAA -3; *AQP9 *(496 bp product): Sense: 5-GGATTTTCAATGGCAGTTGG-3; Antisense: 5-CGGTGAAAAGTCTGGGACTC-3. PCR (30 cycles) was performed (HotStarTaq Master Mix, Qiagen) with 10-20% cDNA and 1 pmol of each primers: hot-start at 95°C for 15 min, denaturation at 95°C for 30 sec, annealing at 56°C to 60°C (dependent on primer optimum) for 30 sec, and elongation at 72°C for 1 min. Negative controls were performed including omission of reverse transcriptase or omission of cDNA. PCR products were separated by 2% agarose gel electrophoresis and the products were photographed under ultraviolet illumination.

### Statistical analysis

All data were analyzed by one-way ANOVA and least significant difference (LSD) post hoc test and were reported as the means ± S.E.M. from five independent observations. Statistical analyses were performed using the Statistica program (StatSoft Inc., Tulsa, USA). Values for *P *< 0.05 were considered statistically significant.

## Results

### Western blot

**Figure 1:**
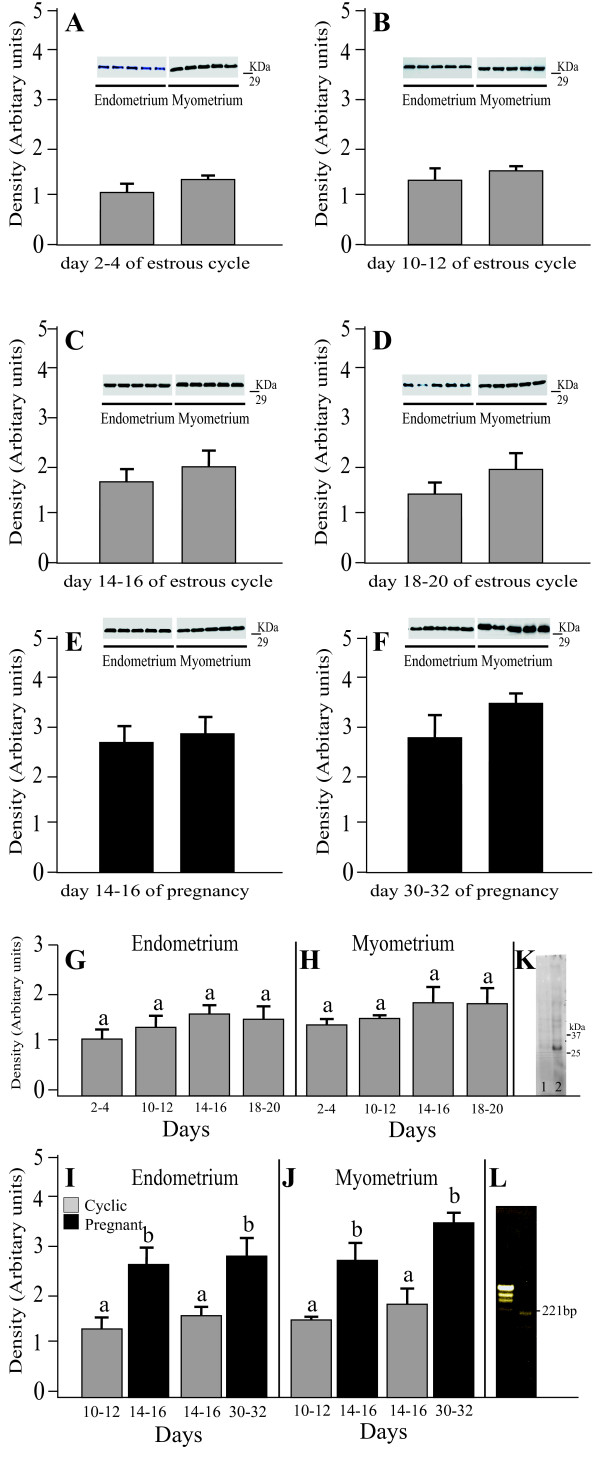
**Western blot analysis of AQP1 expression**. Expression of AQP1 assessed by Western blotting in the endometrium and myometrium of porcine uterus on Days 2-4 (A), 10-12 (B), 14-16 (C) and 18-20 (D) of estrous cycle as well as on Days 14-16 (E) and 30-32 (F) of pregnancy. Densitometric analyses of AQP1 protein levels normalized against β-actin (A-F). The data are summarized in (G) for endometrial and myometrial (H) expression of AQP1 during studied stages of the cycle. Comparison of AQP1 expression determined by Western blotting in porcine endometrium (I) and myometrium (J), between two stages of the cycle and two periods of pregnancy. Immunoblotting for AQP1 protein (K) in the uterus. The blots were probed with anti-AQP1 (Lane 2) or anti-AQP1 preadsorbed with the excess synthetic peptide (Lane 1). RT-PCR was performed on total RNA (L) from uteral tissue. Values are the mean ± SEM (n = 5). ^a,b^Means with different superscripts are significantly different.

The endometrial and myometrial expression of AQP1 protein in the porcine uterus at different stages of the estrous cycle and pregnancy (see "Materials and methods") is shown in Fig. 1A-F and summarized in Fig. 1G-J. A band of AQP1 protein product of the expected size (29 kDa) was clearly detected in all stages of the estrous cycle and pregnancy (Fig. 1A-F). The antibody recognized a clear solitary band with a mobility corresponding to predicted molecular mass of 29 kDa in the porcine uterus (Fig. 1H, Lane 2). The bands were fully ablated by the antibody preadsorbed with the excess immunizing peptide (Fig. 1H, Lane 1). RT-PCR analysis, performed with AQP1-specific primers, yielded ~221-bp DNA product in the uterus (Fig. 1K). Using immunoblotting with the AQP1 antibody, AQP1 was detected in a protein fraction isolated from the porcine kidney, which served as a positive control for AQP1 protein expression (data not shown). In cyclic gilts, endometrial and myometrial expression of AQP1 protein did not change significantly during the various stages of the estrous cycle (Fig. 1G and 1H). Similarly, there was no change in the AQP1 expression within the pregnancy (Fig. 1I and 1J). However, in pregnant gilts, endometrial and myometrial expression of AQP1 increased significantly (*P *< 0.01) on Days 14-16 and on Days 30-32 when compared to the Days 10-12 and 14-16 of the estrous cycle, respectively (Fig. 1I and 1J).

**Figure 2:**
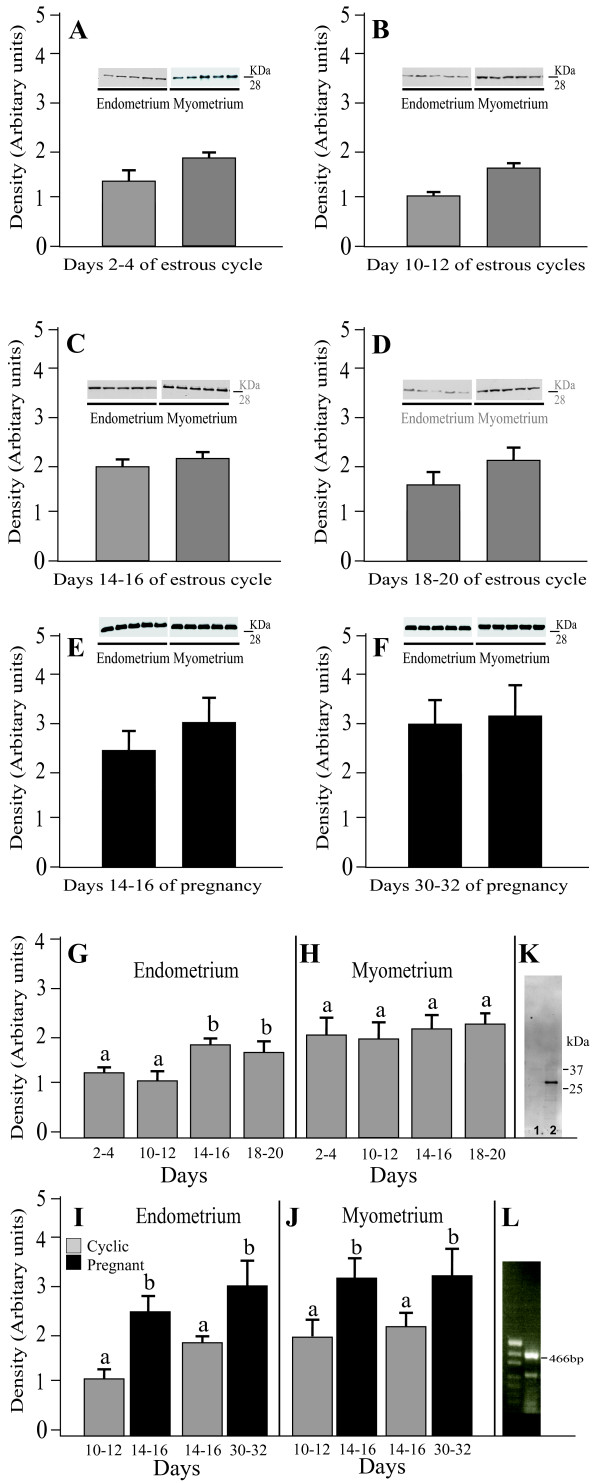
**Western blot analysis of AQP5 expression**. Expression of AQP5 assessed by Western blotting in the endometrium and myometrium of porcine uterus on Days 2-4 (A), 10-12 (B), 14-16 (C) and 18-20 (D) of estrous cycle as well as on Days 14-16 (E) and 30-32 (F) of pregnancy. Densitometric analyses of AQP5 protein levels normalized against β-actin (A-F). The data are summarized in (G) for endometrial and myometrial (H) expression of AQP5 during studied stages of the cycle. Comparison of AQP5 expression determined by Western blotting in porcine endometrium (I) and myometrium (J), between two stages of the cycle and two periods of pregnancy. Immunoblotting for AQP5 protein (K) in the uterus. The blots were probed with anti-AQP5 (Lane 2) or anti-AQP5 preadsorbed with the excess synthetic peptide (Lane 1). RT-PCR was performed on total RNA (L) from uteral tissue. Values are the mean ± SEM (n = 5). ^a,b^Means with different superscripts are significantly different.

The expression of AQP5 in the porcine uterus at different stages of the estrous cycle and pregnancy is shown in Fig. 2A-F and summarized in Fig. 2G-J. A band of AQP5 protein product of the expected size (28 kDa) was clearly detected in all stages of the estrous cycle and pregnancy (Fig. 2A-F). The antibody recognized a clear solitary band with a mobility corresponding to predicted molecular mass of 28 kDa in the porcine uterus (Fig. 2H, Lane 2). The bands were fully ablated by the antibody preadsorbed with the excess immunizing peptide (Fig. 2H, Lane 1). RT-PCR analysis, performed with AQP1-specific primers, yielded ~466-bp DNA product in the uterus (Fig. 2K). Immunoblotting with the anti-AQP5 antibody recognized AQP5 in porcine lung samples, which served as a positive control for AQP5 (data not shown). In cyclic gilts, endometrial expression of AQP5 did not change significantly between Days 2-4 and 10-12 but increased (*P *< 0.05) on Days 14-16 and 18-20 when compared with Days 2-4 and 10-12 of the cycle (Fig. 2G). The myometrial expression of AQP5 level did not differ significantly during the estrous cycle (Fig. 2H). In endometrium and myometrium, expression of AQP5 protein increased (*P *< 0.05-0.01) during the examined stages of pregnancy in comparison to the mid- and late-luteal phases of the estrous cycle (Fig. 2I and 2J). Endometrial and myometrial expression of AQP5 protein did not change significantly during the pregnancy (Fig. 2I and 2J).

**Figure 3:**
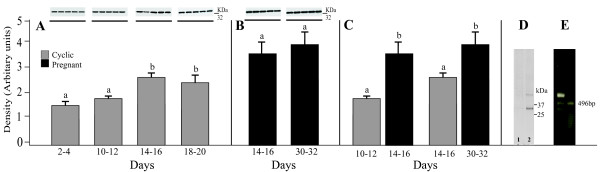
**Western blot analysis of AQP9 expression**. Expression of AQP9 assessed by Western blotting in the endometrium of porcine uterus on Days 2-4, 10-12, 14-16 and 18-20 of estrous cycle (A) as well as on Days 14-16 and 30-32 of pregnancy (B). Densitometric analyses of AQP9 protein levels normalized against β-actin (A and B). The data are summarized for endometrial expression of AQP9 during studied stages of the cycle and pregnancy (A and B). Comparison of AQP9 expression determined by Western blotting in porcine endometrium between two stages of the cycle and two periods of pregnancy (C). Immunoblotting for AQP5 protein (D) in the uterus. The blots were probed with anti-AQP5 (Lane 2) or anti-AQP5 preadsorbed with the excess synthetic peptide (Lane 1). RT-PCR was performed on total RNA (E) from uteral tissue. Values are the mean ± SEM (n = 5). ^a,b^Means with different superscripts are significantly different.

The expression of AQP9 in the porcine uterus at different stages of the estrous cycle and pregnancy is shown in Fig. 3A and B and summarized in Fig. 3C. A band of AQP9 protein product of the expected size (32 kDa) was clearly detected in all stages of the estrous cycle and pregnancy (Fig. 3). The antibody recognized a clear solitary band with a mobility corresponding to predicted molecular mass of 32 kDa in the porcine uterus (Fig. 3D, Lane 2). The bands were fully ablated by the antibody preadsorbed with the excess immunizing peptide (Fig. 3D, Lane 1). RT-PCR analysis, performed with AQP1-specific primers, yielded ~496-bp DNA product in the uterus (Fig. 3E). Immunoblotting with the anti-AQP9 antibody recognized AQP9 in porcine liver samples, which served as a positive control for AQP9 (data not shown). In cyclic gilts, endometrial expression of AQP9 did not change significantly between Days 2-4 and 10-12 but increased (*P *< 0.05) on Days 14-16 and 18-20 when compared to the early and middle stages of the luteal phase (Fig. 3A). Endometrial expression of AQP9 protein did not change significantly during the pregnancy (Fig. 3B). However, in the endometrium, expression of AQP9 increased (*P *< 0.05-0.01) during the onset and the end of implantation process in comparison to the mid- and late-luteal phases of the estrous cycle (Fig. 3C). In the myometrium, the expression of AQP9 was not detected in any stages of the estrous cycle or pregnancy.

Negative controls for immunoblot analyses were performed by omitting or replacing the primary antibodies against AQP1, 5 and 9 with non-immunized rabbit immunoglobulins; no immunostaining was observed (data not shown).

### Immunohistochemistry

**Figure 4:**
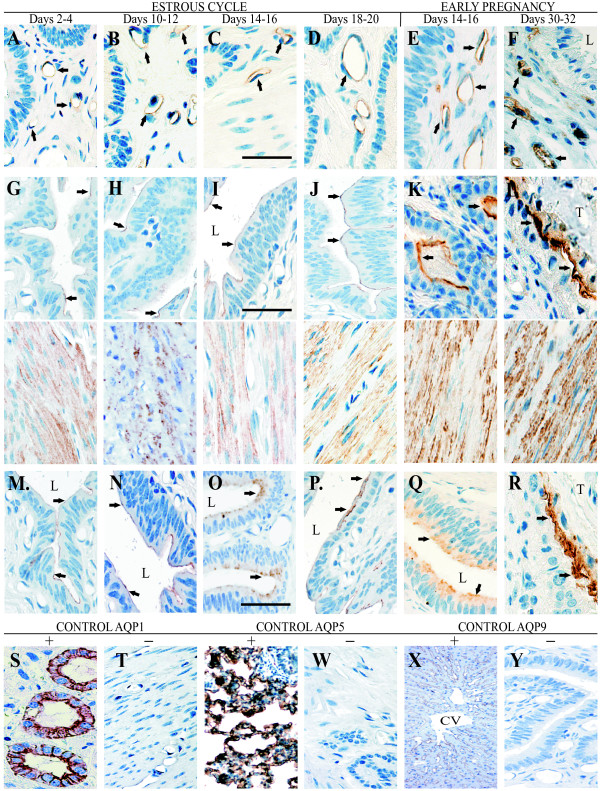
**Immunolocalization of AQP1, 5 and 9**. Immunohistochemical staining of AQP1 in paraffin-embedded sections of the uterus from pigs. Anti-AQP1 antibody labels capillary endothelium of the uterus (A-F). Immunoperoxidase labeling of AQP1 from the pig kidney cortex (positive control) (S). The labeling is seen in both of the apical cell membrane and in the basolateral cell membrane in proximal tubule cells. AQP5 antibody stains luminal and glandular epithelial cells (arrows) and smooth muscle cells (G-L). The anti-AQP5 labels apical membrane of type I pulmonary epithelial cells of the pig (positive control) (U). AQP9 labels luminal and glandular epithelial cells of the uterus (arrows) (M-R). Immunoperoxidase labeling of AQP9 from the pig liver (positive control) (X). No staining was observed with using non-immune immunoglobulins (negative controls) (T, W and Y). L, lumen; CV, central vein; T, trophoblast. Bar = 50 μm.

As stated above, immunohistochemical analysis confirmed expressions of AQP1, AQP5 and AQP9 in the porcine uterus throughout all examined reproductive stages. AQP1 immunoreactivity was detected in the capillary endothelium of the uterus (Fig. 4A-F). As a positive control, AQP1 labeling was seen in the apical and basolateral plasma membranes of the proximal tubule cells in the pig kidney (Fig. 4S), which is consistent with previous findings [26]. AQP5 was localized in smooth muscle cells and luminal and glandular epithelial cells (Fig. 4G-L). Consistent with previous reports [23], the AQP5 antibody noticeably stained (Fig. 4U) the apical membrane of type I porcine pulmonary epithelial cells as a positive control. The anti-AQP9 antibody, similar to AQP5, labeled luminal and glandular epithelial cells of the uterus (Fig. 4M-R). As a positive control, AQP9 staining was seen at the sinusoidal surfaces of hepatocyte plates in the pig liver (Fig. 4X), which is in agreement with a previous observation [27].

Negative controls for all immunohistochemical analyses were performed by omitting (data not shown) or replacing the primary antibodies against AQPs with non-immunized rabbit immunoglobulins; no immunostaining was observed (Fig. 4T, W and 4Y).

## Discussion

The study was undertaken to extend our knowledge about the expression of AQP1, AQP5 and AQP9 protein in porcine uterine tissues during the estrous cycle and early pregnancy. The results demonstrate that protein levels for the studied AQPs varied in distinct tissues depending on the phase of the estrous cycle and the stages of early pregnancy. Using immunoperoxidase and immunoblotting techniques, AQP1, AQP5, and AQP9 were clearly detected in all studied stages of the estrous cycle and early pregnancy.

AQP1 was localized within uterine endometrial and myometrial blood vessels. In cyclic gilts, endometrial and myometrial expression of AQP1 did not change but significantly increased during pregnancy. AQP5 was detected in smooth muscle cells and luminal and glandular epithelial cells. Endometrial expression of AQP5 protein did not change significantly between early-luteal and mid-luteal phases of the estrous cycle but increased on Days 14-16 and 18-20 of the estrous cycle as well as during early pregnancy. Myometrial expression of AQP5 did not change significantly during the estrous cycle but increased in the pregnancy. In contrast, the anti-AQP9 antibody labeled only endometrial luminal and glandular epithelial cells. Endometrial expression of AQP9 did not change significantly between Days 2-4 and 10-12 of the estrous cycle but increased on the late-luteal and late-follicular phase as well as during early pregnancy. So far, there is no available data describing porcine AQP1, AQP5 and AQP9 localization and changes in protein expression at different stages of the estrous cycle and during early pregnancy.

The localization of AQP1 in the uteral capillaries is a reasonable result while the presence of AQP5 in the smooth muscle is unusual in view of its distribution in other organs. However, Helguera et al. [28] and Girotti and Zingg [29] reported AQP5 and AQP8 expression in rat myometrium by microarray and QRT-PCR or by microarray, respectively. Helguera et al. [28] showed a significant downregulation of AQP5 by about ~45- to ~100-fold (determined by QRT-PCR and microarray, respectively) and AQP8 by ~7-fold during delivery, underscoring their potential role in parturition. Moreover, both AQP5 and AQP8 were upregulated during the first 20 Days of pregnancy by 20- and 6-fold, respectively. However, both AQPs were downregulated from Day 20 to Day 23 of pregnancy (~4-fold for AQP5 and ~2-fold for AQP8) [28,29]. There was no change in the expression of AQP5 when comparing term versus labor, whereas AQP8 was further downregulated in labor [29]. Helguera et al. [28] observed similar downregulation of AQP8 in rat myometrium from Day 21 to labor. However, the dramatic downregulation of AQP5 (~100-fold in microarray data and ~45-fold in QRT-PCR) between late pregnancy and labor [28] is not in agreement with the study of Girotti and Zingg [29]. The authors suggested, that this discrepancy could be due to slight differences in the selected time points or to the differences in the tissue composition of uterus that was contaminated with endometrium [29]. In the present study, AQP1, -5, and-9 protein expression in the pig uterus significantly increased during early pregnancy when compare to the estrus cycle. However, expression of above mentioned AQPs in the pig oviduct did not differ when comparing periods of pregnancy versus mid- and late-luteal phases (unpublished data). Frigeri et al. [30] found that expression of sarcolemmal AQP4 together with that of vascular AQP1 may be responsible for the fast water transfer from the blood into the muscle during intense activity. These data imply an important role for AQPs in skeletal muscle physiology as well as an involvement of AQP4 in the molecular alterations that occur in the muscle of DMD patients. It is reasonable to speculate that in the pig uteral smooth muscle AQP5 may act as the AQP4 in the rat skeletal muscle.

The present results clearly demonstrated that AQP1, -5, and -9 protein expression in the porcine uterus is influenced by estrus cycle and pregnancy. The hormonal status of the pig may also influence the luteinizing hormone/human chorionic gonadotropin LH/hCG receptor expression levels in the porcine uterus. The expression of LH/hCG receptors in the myometrium is higher in the progesterone-dominated phase of the estrous cycle than in the estrogen-dominated phase [31]. Moreover, cyclic expression patterns of LH/hCG receptors in the porcine estrous cycles, along with the known effects of estradiol and progesterone on myometrial LH/hCG receptor expression in ovariectomized pigs [32] may suggest that estradiol directly up-regulates and progesterone acts through estradiol-primed tissue to increase LH/hCG receptor levels in the myometrium as well as in the endometrium [33]. The presence of LH/hCG receptors in the myometrium raises an intriguing possibility that LH/hCG may also have a direct effect on uterine function in addition to its indirect action via ovarian steroid hormones. There are data suggesting that LH/hCG receptors may play a role in the hyperplasia and hypotrophy of the uterus [34] and in the uterine motility having an inhibitory effect on the frequency and amplitude of spontaneous myometrial contractions in the porcine uterus [35]. Slattery et al. [36] clearly demonstrated that hCG exerts a significant concentration-dependent relaxant effect on human myometrial tissue obtained in late pregnancy. This inhibitory effect was not estrogen-dependent as occurred in other tissue types. The authors suggest, that these findings clearly demonstrate that hCG has a relaxing effect on the myometrium and thus its clinical utility in the treatment of preterm labor should be estimated. An higher concentration of these receptors in the porcine endometrial tissue was observed on days 10 and 15 of the estrous cycle than on days 5 and 19 [33]. Expression of LH/hCG receptors in the endometrium and endometrial blood vessels of the pig was also demonstrated using autoradiography with 125I-labeled hCG [37]. The higher level of endometrial LH/hCG receptors before and at the time of luteolysis in pigs suggested the possible role of LH in PGs synthesis. Indeed, LH is able to stimulate PGF2 secretion from endometrial tissue [33] as well as separately cultured stromal and luminal epithelial cells [38]. Incubation of endometrial explants with LH resulted in an increase of PGF2 output in a dose-dependent manner on days 5-16 of the estrous cycle. However, the strongest effect was found during luteolysis (days 14-16) of the estrous cycle. Moreover, a LH stimulated upregulation of COX protein has also been shown [33]. Recently, Marino et al. [39] found that hCG may increase AQP9 protein expression and functionality in human preeclamptic placenta via cAMP pathways. They speculate that as a neutral solute channel, AQP9 may be implicated in energy metabolism, or may participate in the clearance of excess lactate in the extracellular space during placental ischemia in preeclamptic placentas.

In the rat kidney, the expression of AQP2 can be regulated by PGs, NO, and COX-2 [40,41]. Recently, nitric oxide synthase 3 (NOS3) was localized in the myometrial and epithelial tissues as well as in blood vessels indicating a contribution of this vasoactive peptide to the uterine imbibition processes [18]. However, further studies are required to substantiate the hypotheses of LH, PGs and NO roles in regulation of AQPs in the uterus.

Two separate experiments performed by Li et al. [6,7] found the presence of AQP-1 mRNA in the human and rat uterus and revealed its regulation by estrogen (E_2_) in the rat. In 2003, Jablonski et al. [14] showed that AQP1 expression was restricted to smooth muscle cells of the mouse myometrium and slightly affected by ovarian steroid hormones. Our immunohistochemical results did not show AQP1 staining in the smooth muscle cells but indicated its presence in the myometrial vessels, which is consistent with our previous studies performed with cyclic pigs [11]. However, recently Aralla et al. [18] demonstrated the expression of AQP1 in the uterine blood vessels and also in the myometrium of cycling bitches. Moreover, in this study, the AQP1 localization and expression were not affected by stages of the estrous cycle [18], like in the pig, as demonstrated in the present study. The estrous cycle stage-independed AQP1 expression in human endometrial vessels was recently confirmed by Mints et al. [42] and Hildenbrand et al. [43]. Hildenbrand et al. [43] demonstrated stronger AQP1 expression in capillaries and arteries than in veins. In addition, Mints et al. [42] demonstrated that the expression of AQP1 in endometrial blood vessels in the menorrhagia group was significantly lower than in controls.

In contrast to AQP1, the endometrial AQP5 and AQP9 expression increased during the luteal phase and reached the highest values on Days 14-16 of the estrous cycle. Our previous study showed the expression of AQP5 and AQP9 in uterine epithelia cells in the late-follicular phase [11]. Aralla et al. [18] demonstrated that elevated expression of AQP5 in the apical plasma membrane of uterine epithelial cells coincides with concentrations of progesterone (P_4_) in plasma. The increased endometrial expression of AQP5 and AQP9 in the late luteal phase may be connected to structural changes in porcine endometrium which occur during this stage of the estrous cycle (for review see [44,45]).

In the current study, the myometrial layer of the porcine uterus exhibited expression of AQP1 and AQP5, which did not change significantly during the estrous cycle. It seems that the relatively stable expression of AQP1 and AQP5 reflects the lack of marked histological changes in this layer during the cycle. Thilander et al. [46] did not observe any major variations in the ultrastructure of the uterine muscle cells throughout the porcine estrous cycle.

Uterine expression of several other AQPs was demonstrated in different species during the estrous cycle. In humans, AQP2 expression has been found in endometrial cells of the uterus [19,20]. The expression of human endometrial AQP2 is stage of menstrual cycle dependent and reaches a higher level at the mid-secretory phase than during the proliferative and early secretory phases [19]. The endometrial AQP2 level was positively correlated to serum E_2 _and P_4 _concentrations [19]. Aralla et al. [18] also revealed the AQP2 immunoreactivity in the uterus of bitches. Moreover, Jablonski et al. [14] documented the presence of AQP2, 3 and 8 in mouse uterine tissues and described up-regulation of AQP2 by E_2. _The authors suggested that, besides AQP1, AQP2, AQP3 and AQP8 might participate in water movement during uterine imbibition [14]. Additionally, AQP2 and AQP3, due to localization in luminal epithelial cells, are involved in water movement into the lumen of the uterus [14].

As far as early pregnancy is concerned, the present study generally demonstrated increased expression of all studied AQPs (AQP1, 5 and 9) in the uterus during this period in comparison to the estrous cycle. Richard et al. [15] showed distinct uterine expression patterns for AQP1 and AQP5 in pregnant mice. AQP1 was localized to the inner circular myometrium throughout the peri-implantation period, which is in agreement with the study by Lindsay and Murphy [47], who found increasing contents of this AQP in rat uterus from Day 1 to Day 6 of pregnancy. Besides the myometrial localization, AQP1 was found in endothelial cells of the endometrium with maximal protein expression in response to combined treatment with P_4 _and E_2 _[16]. The present study did not confirm the localization of AQP1 in the myometrium but in the uterine vessels of pregnant gilts. In turn, AQP5 expression was observed in the glandular epithelium (the basolateral region) of the mouse uterus during early pregnancy with a marked increase on Day 5 [15]. On the other hand, Lindsay and Murphy [16] found the stimulation of AQP5 protein expression in the apical plasma membrane of rat uterine epithelial cells by P_4 _alone or in combination with estradiol, to a level similar to that observed during implantation. Lindsay and Murphy [16] also found that the redistribution of AQP5 ranged from completely cytoplasmic in early pregnancy to predominantly apical plasma membrane localization at the time of implantation. The authors suggest that this redistribution of AQP5 may be involved in peri-implantation fluid homeostasis. In the present study, AQP5 and AQP9 were found on the apical plasma membrane of the epithelial cells and their expressions were increased during both stages of implantation. Recently, the presence of mRNA for several AQP isoforms (AQP1, 5, 7, 8 and 9) was demonstrated in the rat uterus at the time of implantation while the immunohistochemical study confirmed an apical distribution of AQP5 and 9 in the glandular epithelium during this period [17]. The authors suggest that this localization provides a mechanism for transcellular fluid transport through these cells similar to that in luminal epithelial cells reported in previous papers [47,48]. Moreover, Richard et al. [15] described uterine expression of AQP4, which appeared to be abundant in the luminal epithelium in mice on Day 1 of pregnancy in mice but barely detectable at the time of implantation. It should be noted that in porcine smooth muscle cells only increased AQP5 concentration was seen during early pregnancy. This observation is in contrast to those reported for rodents, in which AQP1 was found in this tissue [15,47].

In conclusion, the present study provides an anatomical basis for AQP1, 5 and 9 expressions in the porcine uterus during different stages of the estrous cycle and pregnancy. The uterine expression of these proteins remains generally low in cyclic gilts while it markedly increases in early pregnancy. Aquaporins play important functions in the regulation of water movement in different tissues and therefore they may contribute to creating an uterine environment that enables embryonic attachment and survival in pregnant gilts. Further studies are needed to determine precisely the role of AQP molecules in the establishment of an optimal uterine condition for developing conceptus.

## Competing interests

The author declares that they have no competing interests.
